# Molecular characterization and genotype of multi-drug resistant *Staphylococcus epidermidis* in nasal carriage of young population, Mahasarakham, Thailand

**DOI:** 10.17305/bb.2024.11116

**Published:** 2024-10-08

**Authors:** Peechanika Chopjitt, Panita Tangthong, Jiranuch Kongkaem, Pritprapoan Wonkyai, Achira Charoenwattanamaneechai, Surasak Khankhum, Phitcharat Sunthamala, Anusak Kerdsin, Nuchsupha Sunthamala

**Affiliations:** 1 Faculty of Public Health, Kasetsart University Chalermphrakiat, Sakon Nakhon Campus, Sakon Nakhon, Thailand; 2 Department of Biology, Faculty of Science, Mahasarakham University, Mahasarakham, Thailand; 3 Mahasarakham University Demonstration School (Secondary), Mahasarakham University, Mahasarakham, Thailand; 4 Department of Biotechnology, Faculty of Technology, Mahasarakham University, Mahasarakham, Thailand

**Keywords:** Antimicrobial resistance gene, nasal carriage, *Staphylococcus epidermidis*, multi-drug resistant, biofilm

## Abstract

*Staphylococcus epidermidis*, a coagulase-negative staphylococcus, is a prevalent skin commensal that has increasingly been recognized as a significant pathogen, particularly in hospital environments, where it is associated with device-related infections. The emergence of multi-drug resistance and its ability to form biofilms complicate the clinical management of infections caused by this organism, posing a growing public health concern. This study aimed to investigate the nasal carriage of *S. epidermidis* among healthy young individuals and to analyze its antibiotic resistance patterns, resistance genes, and biofilm formation capabilities. Nasal swabs were collected from 40 undergraduate students at Mahasarakham University, Thailand, aged between 20 and 22 years. A total of 38 isolates were confirmed as *S. epidermidis* through both phenotypic and molecular characterization. Antibiotic susceptibility testing demonstrated resistance to various classes of antimicrobials, including beta-lactams, macrolides, and tetracyclines. Notably, five isolates exhibited methicillin resistance *S. epidermidis* (MRSE). Resistance genes, such as *mecA*, *ermA*, *tetM*, *tetL*, and *tetK*, were identified across the isolates, contributing to the observed resistance profiles. Biofilm formation assays revealed that most isolates displayed weak to moderate biofilm formation, with only one isolate demonstrating strong biofilm-forming capacity. Genetic analysis indicated a significant correlation between biofilm formation and the presence of the *icaA* gene, which is crucial for biofilm production. This study suggests the necessity for ongoing surveillance of nasal carriage of *S. epidermidis* to enhance understanding of its role in the dissemination of antimicrobial resistance and biofilm-associated infections, particularly within healthcare settings.

## Introduction

Staphylococcus spp., although part of the normal human skin microbiota, are capable of causing a wide range of infections, including those affecting the urinary and respiratory tracts, wounds, soft tissues, blood, and even the heart (endocarditis) [1, 2]. *Staphylococcus epidermidis*, a Gram-positive, non-spore-forming, non-motile, facultative anaerobe that is catalase-positive and coagulase-negative, is recognized as a significant cause of hospital-acquired infections, particularly those related to medical devices [3, 4]. Notably, *S. epidermidis* accounts for approximately 13% of prosthetic valve endocarditis cases, often involving intracardiac abscesses in about 38% of cases and associated with a high mortality rate of 24% [5]. The rise of antibiotic-resistant strains of *S. epidermidis* poses a substantial challenge in healthcare settings. These resistant strains are responsible for an estimated 100,000 infections annually in the United States, contributing to a mortality rate of around 10% [6–8].

Nasal carriage of *S. epidermidis* serves as a reservoir for potential infections, especially in individuals with compromised immune systems or those undergoing surgical procedures. The bacterium’s ability to produce surface proteins and exopolysaccharides enhances its colonization of the nasal mucosa and facilitates infections by promoting adherence to nasal epithelial cells and evading immune responses [9]. Coagulase-negative staphylococci (CoNS) isolated from various healthcare facilities show resistance to multiple classes of antibiotics, including tetracyclines, aminoglycosides, cephalosporins, fluoroquinolones, penicillins, and macrolides [6–8, 10]. This resistance is linked to various genes, including *mecA*, *aacA-D*, *tetK*, *tetM*, *ermA*, *ermC*, *msrA*, *msrB*, *linA*, *vatA*, *vatB*, and *vatC* [2, 6–8]. The *mecA* gene, found on the staphylococcal cassette chromosome *mec* (SCCmec), is particularly crucial for methicillin resistance and can be horizontally transferred between species, helping spread methicillin-resistant *Staphylococcus aureus* (MRSA) [9, 11]. Tetracycline resistance is also common among staphylococci [12, 13]. Linezolid (LNZ) has emerged as a promising alternative to vancomycin, particularly in intensive care unit (ICU) settings, due to its favorable safety profile and efficacy. However, resistance mechanisms are being increasingly recognized, including mutations in the 23S rRNA gene, acquisition of the *cfr* gene, and mutations in ribosomal proteins L3 and L4 [14].

In a tertiary children’s hospital in Cracow, Poland, the emergence of linezolid-resistant *S. epidermidis* (LRSE) poses a serious threat. Genetic characterization of 11 LRSE isolates collected between 2015 and 2017 revealed multi-drug resistance (MDR), biofilm formation capabilities, and distinct SCCmec cassette compositions [15]. Similarly, a case-control study conducted in a French surgical ICU from 2012 to 2016 identified 13 cases of LRSE, which were linked to prior linezolid exposure, prolonged ICU stays, and high Charlson comorbidity scores. Pulsed-field gel electrophoresis (PFGE) and multilocus sequence typing (MLST) showed clonal spread among LNZ-resistant isolates [16]. The spread of resistance in staphylococci is primarily driven by plasmids and transposons that enable the transfer of resistance genes across different species and genera [17–23].

*S. epidermidis* possesses several virulence factors, including toxic shock syndrome toxin-1 (TSST-1), exfoliative toxins A and B, clumping factor (ClfA), and various types of the accessory gene regulator (agr). The X-region gene, which plays a key role in disease occurrence, varies, allowing for differentiation during outbreak investigations, while the IgG-binding region contributes to host specificity and immune response modulation. Both regions are frequently found in staphylococcal infections [1, 24]. One of the key virulence factors of *S. epidermidis* is its ability to form biofilms, primarily composed of polysaccharide intercellular adhesin (PIA). Biofilm formation is linked to antimicrobial resistance due to reduced drug penetration and is more common in invasive isolates compared to those from healthy individuals [25–28]. This biofilm-forming capability is also observed in other staphylococci, such as *S. aureus* [29]. PIA production is facilitated by an enzyme encoded by the ica operon, which includes the genes *icaA*, *B*, *C*, and *D*, as well as the transposable element IS256 [30]. Specifically, *icaA* encodes the enzyme responsible for PIA synthesis, while *icaD* enhances this activity. *icaB* deacetylates mature PIA, and *icaC* is likely involved in the externalization and elongation of the polymer [27, 31]. Regulation of the *icaADBC* genes is complex and is influenced by factors, such as the insertion of IS256 into various parts of the operon, accounting for up to 33% of operon activation [28, 32–34]. Other regulatory genes, including *rsbU*, *sB*, *tcaR*, *agr*, and *sarA*, also contribute to PIA production [35]. Biofilm formation occurs in two stages: initial surface adhesion, mediated by adhesive polysaccharides and proteins such as autolysins, and cell accumulation, driven by PIA synthesis following ica operon activation [27]. The presence of *icaADBC* genes has been linked to persistent infections and treatment failures involving medical devices. Researchers are exploring the potential of these genes as prognostic markers for device-associated infections [36]. Additionally, biofilm regulation is influenced by various environmental conditions and staphylococcal phenotypes [25].

The aim of this research was to analyze the antimicrobial resistance profiles and biofilm-forming capabilities of *S. epidermidis* isolated from the nasal cavities of healthy young individuals. The study involved undergraduate students enrolled in the Microbiology program at the Faculty of Science, Mahasarakham University, Thailand. These students regularly work with bacterial cultures and antimicrobial agents as part of their academic training. Therefore, assessing nasal carriage of *S. epidermidis* in this population may reveal distinct patterns of antimicrobial resistance compared to other studies. Specific primers were used to collect and identify nasal carriage Staphylococcus species, and the study evaluated antimicrobial susceptibility alongside resistance genes. Biofilm formation, a key bacterial virulence factor, was assessed both phenotypically and through the analysis of biofilm-associated genes. Understanding the mechanisms underlying resistance and virulence in MDR *S. epidermidis* is crucial for mitigating the impact of this opportunistic pathogen on public health.

## Methods

### Sample collection

**Figure 1.**
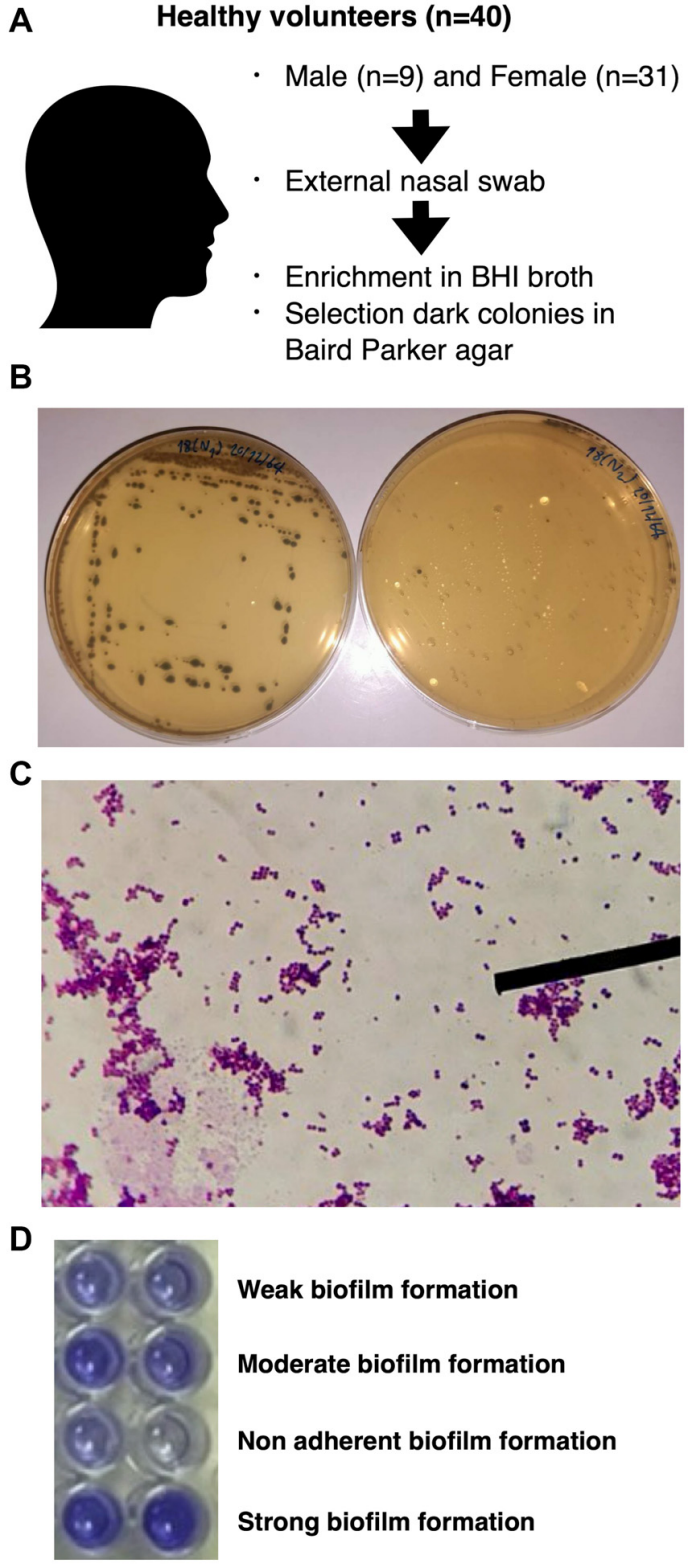
**Sample collection and bacterial characteristics.** (A) The nasal swab samples were collected, and bacteria were enriched in BHI broth; (B) The dark colonies of selected bacteria on Baird Parker agar; (C) Gram staining and microscopic inspection of single colony; (D) The biofilm-forming capacity was classified into four categories: non-adherent (less than 1), weak (1–2), moderate (2–4), and strong (more than 4). BHI broth: Brain heart infusion broth.

Swab samples were collected from the external nasal cavity of healthy volunteers aged 20–22 years, following the acquisition of informed written consent. Each swab was immediately placed into Brain Heart Infusion Broth (BHI Broth, Himedia, India), supplemented with 0.01% w/v potassium tellurite, and incubated under aerobic conditions at 37 ^∘^C for 18–24 h. Samples showing dark turbidity were then streaked onto Baird Parker agar (BPA, Himedia, India) and incubated at 37 ^∘^C for 18–24 h. Suspected *Staphylococcus spp.* colonies were isolated and stored at −20 ^∘^C until further processing for molecular characterization. Preliminary phenotypic tests, including microscopic inspection, Gram staining, a catalase production test, and a coagulase tube test using rabbit plasma (Figure 1A–C), were conducted prior to species-level identification through polymerase chain reaction (PCR) using specific primers (Table 1).

### Polymerase chain reaction

Genomic DNA from *Staphylococcus spp.* isolates was extracted using the GF-1 Bacterial DNA Extraction Kit (Vivantis, Malaysia). Briefly, 1 mL of bacterial culture grown for 18–24 h at 37 ^∘^C was centrifuged at 6000×*g* for 2 min at room temperature, and the supernatant was discarded. The cell pellet was resuspended in lysis buffer and treated with 20 µL of lysozyme (50 mg/mL). After centrifugation, proteinase K was added for protein denaturation, and RNA was removed with RNase A. The mixture was homogenized, and genomic DNA was precipitated using absolute ethanol. The resulting mixture was loaded onto a column and centrifuged to discard the flow-through; the column was then washed, dried, and eluted. The bacterial genomic DNA was stored at -20 ^∘^C. The isolates were identified at the species level via PCR, with the reaction mixture comprising a total volume of 25 µL, including 2 µL of DNA, 10× PCR Buffer, 50 mM MgCl_2_, primers (10 µM forward and reverse), 10 mM dNTPs, and one unit of Taq DNA polymerase (Vivantis, Malaysia). Thermal cycling conditions included an initial denaturation at 95 ^∘^C for 5 min, followed by 40 cycles of denaturation at 95 ^∘^C for 15 s, annealing at 50 ^∘^C for 30 s, and extension at 72 ^∘^C for 40 s, concluding with a final extension at 72 ^∘^C for 7 min. PCR amplicons of *Staphylococcus spp.* and *S. epidermidis* were separated on a 1.5% agarose gel (Vivantis, Malaysia), stained with Red Safe (Vivantis, Malaysia), and visualized under UV illumination. Product sizes were approximately 370 base pairs (bp) for *Staphylococcus spp.* and 124 bp for *S. epidermidis* (Table 1) [37]. Positive controls included reference strains *S. aureus* ATCC 25923 and *S. epidermidis* ATCC 49461 (ATCC, VA, USA).

**Table 1:** Specific primers for detection of bacteria, antibiotics-resistant genes, and biofilm-associated genes

**Category**	**Target**	**Primers**	**PCR product size (base pair)**	**References**
Identification bacteria strains	*Staphylococcus* spp.	FW-TIACCATT TCAGTACCTTC TGGTAA RV-GGCCGTGT TGAACGTGGTC AAATCA	370	[37]
	*Staphylococcus epidermidis*	FW-CAAAAGAG CGTGGAGAAAA GTATCA RV-ATCAAAAA GTTGGCGAACC TTTTCA	124	[37]
Antibiotics-resistant genes	*mec*A	FW-AAAATCGA TGGTAAAGGTT GGC RV-AGTTCTGG CACTACCGGAT TTGC	533	[71]
	*tet*K	FW-TTATGGTG GTTGTAGCTAG AAA RV-AAAGGGTT AGAAACTCTTG AAA	348	[72]
	*tet*L	FW-TGGTGGAA TGATAGCCCAT T RV-CAGGAATG ACAGCACGCTA A	229	[73]
	*tet*M	FW-GTGGACAA AGGTACAACGA G RV-CGGTAAAG TTCGTCACACA C	406	[73]
	*erm*A	FW-TCTAAAAA GCATGTAAAAG AA RV-TGATTATT ATTTGATAGCT TC	645	[74]
	*erm*B	FW-TGGTATTC CAAATGCGTAA TG RV-CTGTGGTA TGGCGGGTAAG T	745	[73]
	*erm*C	FW-TCAAAACA TAATATAGATA AA RV-TAACTGCT AAATTTGTTAT AATCG	642	[74]
	*msr*A/B	FW-GCAAATGG TGTAGGTAAGA CAACT RV-TAAAACAA ATGTAGTGTAC TA	399	[74]
	Int (Tn*916*/Tn*1545*)	FW-GCGTGATT GTATCTCACT RV-GACGCTCC TGTTGCTTC	1028	[75]
Biofilm-associated genes	IS*256*	FW-5′-TGAAA AGCGAAGAGAT TCAAAGC-3′ RV-5′-ATGTA GGTCCATAAGA ACGGC-3′	1102	[30, 70]
	*arc*A	AIPS.27-CTA ACACTGAACCC CAATG AIPS.28-GAG CCAGAAGTACG CGAG	1942	[44]
	*opp*3AB	AIPS.45-GCA AATCTGTAAAT GGTCTGTTC AIPS.46-GAA GATTGGCAGCA CAAAGTG	1183	[44]
	*ica*A	FW-TCTCTTGC AGGAGCAATCA A RV-TCAGGCAC TAACATCCAGC A	188	[42]
	*ica*D	FW-ATGGTCAA GCCCAGACAGA G RV-CGTGTTTT CAACATTTAAT GCAA	198	[42]

### PCR detection for resistance genes of S. epidermidis

PCR detection of resistance genes in *S. epidermidis* was conducted following previously described methods. Specific primers targeting antimicrobial resistance genes, such as *mecA*, *tetK*, *tetL*, *tetM*, *ermA*, *ermB*, *ermC*, *msrA/B*, and *int* (Tn916/Tn1545), were used (Table 1). PCR amplicons were separated on 1.5% agarose gels, stained with Red Safe (Vivantis, Malaysia), and visualized under UV illumination [38].

### Antimicrobial susceptibility testing

Antimicrobial susceptibility testing was performed using the disk diffusion method on Mueller–Hinton agar (Himedia, India), following the Clinical and Laboratory Standards Institute (CLSI) guidelines for 2023. Eleven antimicrobial agents from various classes were tested: cefoxitin (CX-30 µg), erythromycin (E-15 µg), clindamycin (CD-2 µg), penicillin (P–10U), trimethoprim/sulfamethoxazole (COT-1.25/23.75 µg), linezolid (LZ-30 µg), ciprofloxacin (CIP-5 µg), tetracycline (CT-30 µg), rifampicin (RIF-5 µg), chloramphenicol (C-30 µg), and gentamicin (CN-10 µg) (Himedia, India). Reference strains *S. aureus* ATCC 25923 and *S. aureus* ATCC 29213 (ATCC, VA, USA) were used as controls to ensure accurate susceptibility testing. Inoculated plates were incubated at 35 ^∘^C for 18–24 h. After incubation, inhibition zones were measured and classified as susceptible, intermediate, or resistant according to CLSI guidelines [39]. Each antimicrobial susceptibility test was performed in triplicate. Additionally, MDR profiles of the isolates were assessed based on criteria from Magiorakos et al. [40].

To detect inducible clindamycin resistance, a D-test was performed as described by Chavez-Bueno et al. [41]. Bacterial isolates were swabbed onto Mueller–Hinton agar at a 0.5 McFarland standard concentration. Clindamycin (2 µg) and erythromycin (15 µg) disks were placed approximately 1.5 cm apart in the center of the agar plate. Plates were incubated at 35 ^∘^C for 24 h. Inducible resistance was indicated by a blunted inhibition zone surrounding the clindamycin disk adjacent to the erythromycin disk.

### Biofilm formation assay

The biofilm formation assay was adapted from Gad et al. [42]. Briefly, bacterial isolates were inoculated in 3 mL of trypticase soy broth (TSB, Himedia, India) supplemented with 1% glucose and incubated aerobically at 37 ^∘^C for 24 h. Bacterial cultures were adjusted to 0.5 McFarland turbidity using TSB before adding the suspension into a 96-well plate containing 100 µL of TSB per well (containing 10^ImEquation2^ CFU/well). The plate was incubated at 37 ^∘^C for 24 and 48 h. After incubation, the medium was removed from the wells, which were then rinsed twice with deionized water before being dried and stained with 200 µL of 0.1% crystal violet. After staining for 10 min and washing twice with deionized water, the wells were air-dried, and 200 µL of absolute ethanol were added, incubating at 25 ^∘^C for 5 min. Then, 100 µL of the staining solution was transferred to another 96-well plate for absorbance measurement at 595 nm using a spectrophotometer. Each isolate was tested in triplicate across three independent experiments. Absorbance values were averaged along with the standard deviation. Mean absorbance values were normalized against the absorbance of the negative control. Biofilm-forming capacity was classified into four categories: non-adherent (less than 1), weak (1–2), moderate (2–4), and strong (greater than 4) (Figure 1D) [43].

**Figure 2.**
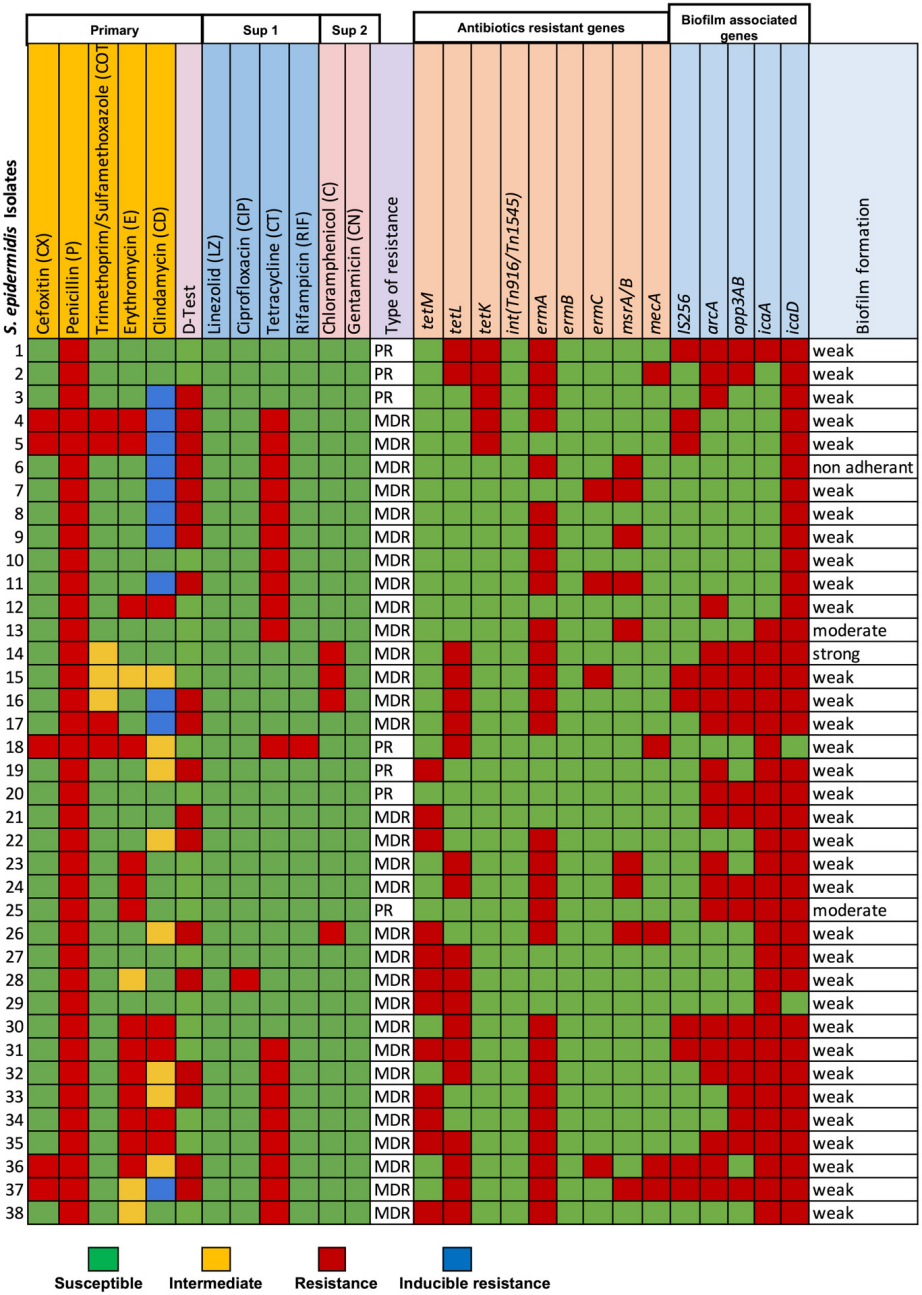
Distribution of antimicrobial resistance and biofilm formation of 38 strains of *Staphylococcus epidermidis* isolates.

### Detection of resistance and biofilm-associated genes of S. epidermidis

PCR analysis targeting biofilm-associated genes in *S. epidermidis* used primers specific to five genes: *IS256*, ACME (*arcA*), opp3 gene clusters (*opp3AB*), intercellular adhesion gene A (*icaA*), and intercellular adhesion gene D (*icaD*) (Table 1). PCR amplicons were separated on 1.5% agarose gels and visualized under UV illumination [30, 38, 44].

### Ethical statement

This study was approved by the Ethics Committee of Mahasarakham University, Thailand (No. 303-221/2023). The research was conducted from August 2022 to August 2023, and informed written consent was obtained from all healthy volunteers aged 20–22 years.

### Statistical analysis

Correlation analyses were performed using Spearman’s correlation coefficient with two-tailed significance testing. *P* values ≤ 0.05 were considered statistically significant, using GraphPad Prism software version 10.

## Results

### Isolation of nasal carriage S. epidermidis

A total of 72 Gram-positive cocci isolates were obtained from 40 healthy volunteers. Among these, 43 isolates were identified as *Staphylococcus* spp. Further analysis using specific primers revealed that 38 of these isolates were specifically identified as *S. epidermidis* (Figure 2).

### Antimicrobial resistance patterns and resistance genes of nasal carriage S. epidermidis isolates

The antimicrobial resistance profile of *S. epidermidis* showed that 5 out of the 38 isolates were resistant to cefoxitin, a marker of methicillin resistance, classifying them as methicillin-resistant *S. epidermidis* (MRSE) (isolates 4, 5, 18, 37, and 38). All isolates demonstrated resistance to penicillin, indicating universal resistance within the sampled population. Resistance or intermediate resistance to trimethoprim/sulfamethoxazole was observed in seven isolates (4, 5, 14, 15, 16, 17, and 18), while the majority remained susceptible. A few isolates (14, 15, 16, and 26) exhibited resistance to chloramphenicol. Notably, there was no observed resistance to linezolid, ciprofloxacin, or gentamicin, suggesting these antibiotics remain effective against the isolates in this study. A significant portion (19 out of 38) displayed resistance to tetracycline, highlighting the prevalence of tetracycline resistance genes within this population. Resistance to rifampicin was rare, with only one isolate (18) showing resistance. However, resistance to erythromycin and clindamycin was widespread among the isolates (Figure 2).

Analysis of antibiotic resistance genes in *S. epidermidis* indicated that the *tetM* gene was the most frequently detected tetracycline resistance gene, present in 12 isolates. The *tetL* gene was found in 19 isolates, while *tetK* was observed less frequently, in only five isolates. The *ermA* and *ermB* genes, which confer resistance to macrolides and lincosamides, were commonly identified; specifically, *ermA* was present in 28 isolates, while *ermB* was absent. The less common *ermC* gene was detected in four isolates. The *mecA* gene, associated with methicillin resistance, was identified in five isolates (2, 18, 26, 36, and 37), confirming their MRSE status (Figure 2).

The antibiotic resistance patterns showed that five MRSE isolates (4, 5, 18, 37, and 38) were resistant to multiple antimicrobials, particularly macrolides (erythromycin and clindamycin) and tetracyclines. These MRSE isolates also harbored the *mecA* gene in four out of the five cases. A substantial portion of the isolates (31 out of 38) exhibited MDR, particularly against penicillin, tetracycline, erythromycin, and clindamycin. These MDR isolates frequently contained both *erm* and *tet* genes (Figure 2).

### Biofilm formation and biofilm-associated genes of nasal carriage S. epidermidis

The biofilm formation capacity among the isolates ranged from weak to moderate, with only one isolate showing strong biofilm formation. This characteristic is clinically relevant, as biofilm-forming bacteria can evade the immune system and resist antimicrobial treatment, complicating infection management. Biofilm formation is associated with specific genetic determinants, such as *icaA* and *icaD*, which were present in varying degrees among the isolated strains (Figure 2).

### Correlation analysis of antimicrobial resistance patterns and biofilm formation of nasal carriage S. epidermidis isolates

**Figure 3.**
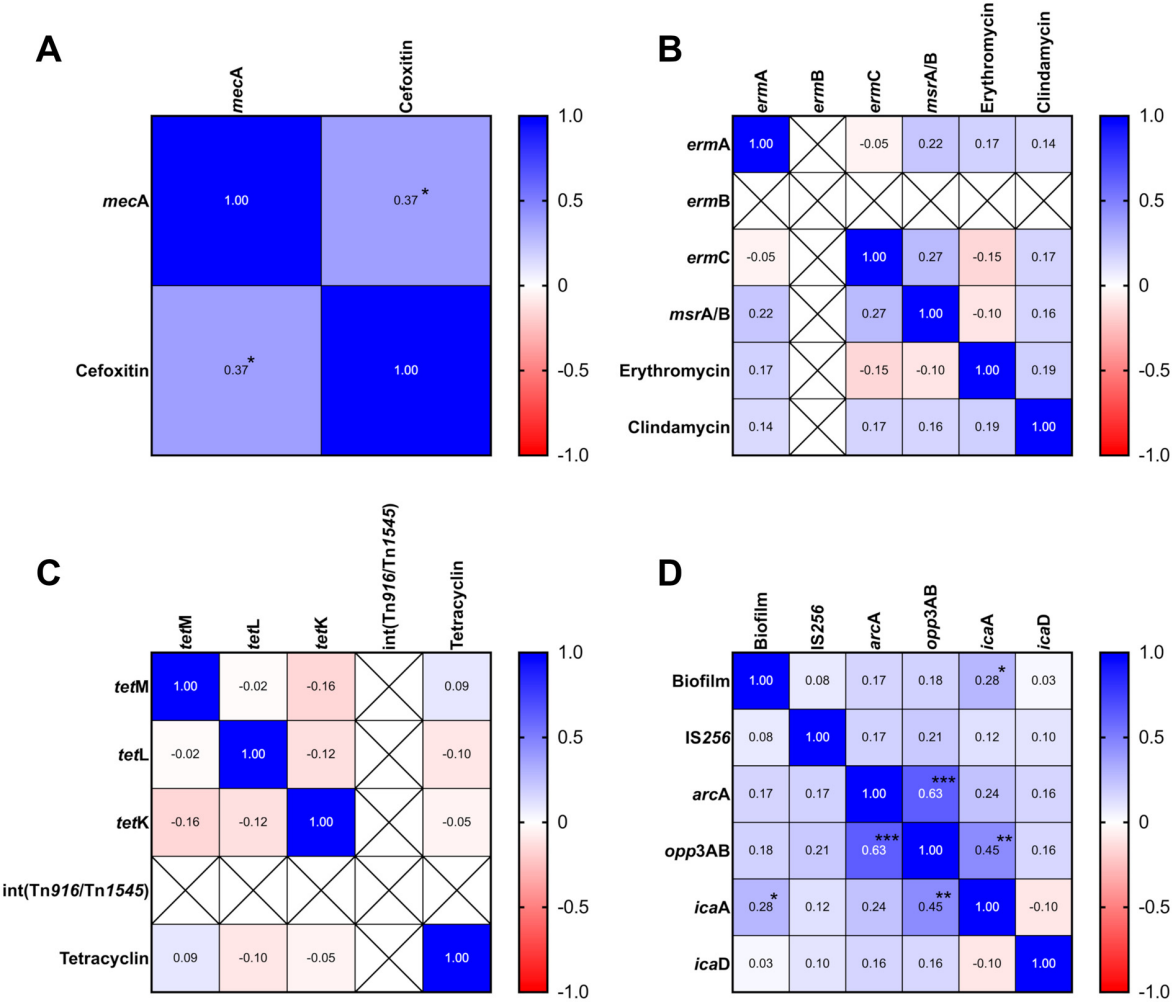
**Correlation analysis of antimicrobial resistance patterns and biofilm formation of nasal carriage *Staphylococcus epidermidis* isolates.** (A) The correlation analysis of cefoxitin resistant and *mec*A genes; (B) The correlation analysis of macrolides and lincosamides resistant with resistant genes; (C) The correlation analysis of tetracycline resistant with resistant genes; (D) The correlation analysis of biofilm formation and biofilm-associated genes. * as *P* ≤ 0.05, ** as *P* ≤ 0.01, and *** as *P* ≤ 0.001.

Correlation analysis between antimicrobial resistance patterns and associated genes revealed a significant positive correlation between cefoxitin resistance and the presence of the *mecA* gene (*r*^2^ ═ 0.37; *P* ≤ 0.05) (Figure 3A). However, no significant correlation was found between macrolide/lincosamide or tetracycline resistance and their respective resistance genes (Figure 3B and 3C). In addition, biofilm formation showed a significant positive correlation with the presence of the *icaA* gene (*r*^2^ ═ 0.28; *P* ≤ 0.05). Moreover, biofilm-associated genes showed significant positive correlations between *arcA* and *opp3AB* (*r*^2^ ═ 0.63; *P* ≤ 0.001) and between *icaA* and *opp3AB* (*r*^2^ ═ 0.45; *P* ≤ 0.01) (Figure 3D).

## Discussion

The nasal carriage of *S. epidermidis* in healthy individuals provides a valuable model for investigating antimicrobial resistance and biofilm formation, both of which are critical factors in the pathogenic potential of this organism. In this study, 38 isolates of *S. epidermidis* were identified from the nasal microbiota of 40 volunteers, indicating a significant prevalence of this species in the nasal cavity. The antimicrobial resistance profile of these isolates revealed extensive resistance to multiple antibiotics, particularly penicillin, with all 38 isolates demonstrating resistance to this agent. This high prevalence of penicillin resistance aligns with global trends, as the widespread presence of β-lactamase enzymes in staphylococci has rendered penicillin ineffective against these organisms [45]. The implications of broad-spectrum β-lactam antibiotic resistance for global healthcare are substantial. This resistance is primarily regulated by the BlaR1 receptor, which, upon detecting β-lactams, activates its metalloprotease domain, resulting in the derepression of the *blaZ* and *mecA* genes, critical for antibiotic resistance. Recent structural analysis using cryo-electron microscopy has elucidated BlaR1’s direct cleavage of the repressor BlaI, without the need for auxiliary components, alongside an essential autocleavage that enhances its capacity to mediate antibiotic resistance [46].

The identification of MRSE in five isolates is particularly concerning, as MRSE infections are notoriously difficult to treat and often require last-resort antibiotics, such as vancomycin or linezolid. Previous studies have reported methicillin resistance rates in *S. epidermidis* ranging from 70% to 92% in certain healthcare institutions [47], frequently accompanied by co-resistance to other antibiotic classes [45, 48]. Interestingly, most isolates in this study remained susceptible to linezolid, ciprofloxacin, and gentamicin, suggesting these antibiotics still retain efficacy against nasal carriage *S. epidermidis*. However, the observation that 19 out of 38 isolates exhibited resistance to tetracycline is notable, given that tetracycline is commonly used to treat mild staphylococcal infections.

Resistance or intermediate resistance to trimethoprim/sulfamethoxazole was observed in 7 of the 38 isolates. The mechanisms underlying resistance to trimethoprim and sulfonamides are multifaceted, involving permeability barriers and efflux pumps that often act synergistically. For example, transposons from the Tn21 family typically link resistance traits to both trimethoprim and sulfonamides, facilitating horizontal transfer among bacterial populations [49]. Resistant organisms like *Pseudomonas aeruginosa*, *Klebsiella pneumoniae*, and *Serratia marcescens* use efflux pumps and permeability barriers [50, 51]. Laboratory studies have frequently identified single amino acid mutations in the chromosomal *dhps* gene of *Escherichia coli*, a phenomenon also observed in clinically relevant bacteria, such as *S. aureus*, *Staphylococcus haemolyticus*, *Campylobacter jejuni*, and *Helicobacter pylori*. In contrast, *Streptococcus pneumoniae* exhibits sulfonamide resistance due to two amino acid duplications in the *folP* gene, altering enzyme structure. For *Streptococcus pyogenes*, rapid sulfonamide resistance is more likely due to transformational recombination rather than sequential mutations [52–54].

Chloramphenicol remained largely effective against *S. epidermidis*, with only 4 of 38 isolates showing resistance, indicating divergent genetic determinants of antibiotic resistance [55].

The genetic analysis of antimicrobial resistance genes provided further insights into the mechanisms driving resistance in these isolates. The identification of the *tetM* gene in 12 isolates and the *tetL* gene in 19 isolates reveals the prevalence of tetracycline resistance genes, aligning with the phenotypic resistance observed. Interestingly, the *tetK* gene and the integron associated with Tn916/Tn1545 were not detected, suggesting divergent evolutionary pathways or selective pressures influencing the acquisition of resistance genes in *S. epidermidis* [56]. Correlation analysis confirmed a significant association between cefoxitin resistance and the presence of the *mecA* gene, which encodes penicillin-binding protein (PBP2a) and serves as the primary determinant of methicillin resistance in staphylococci.

In a study of 151 *S. aureus* isolates from burn patients, 63.6% tested positive for the *mecA* gene, and a significant proportion also harbored tetracycline resistance genes, with 32.4% containing *tetM* and 17.2% *tetK*. Additionally, aminoglycoside-modifying enzyme (AME) genes were identified in 69 isolates, with many exhibiting combinations of multiple AME genes [57]. Recent surveillance data show concerning rates of tetracycline resistance across Europe, with extended-spectrum β-lactamase (ESBL)-producing *E. coli* showing resistance rates of 66.9% and *Klebsiella spp.* at 44.9% [58]. Global statistics reveal a tetracycline resistance rate of 8.7% for MRSA and 24.3% for *S. pneumoniae* [59]. Resistance mechanisms typically involve mobile genetic elements that confer tetracycline-specific genes, along with mutations in ribosomal binding sites and chromosomal alterations that enhance intrinsic resistance [60]. Among the identified tetracycline-specific efflux pumps, group 1 pumps like *tetA* and *tetB* are most relevant in Gram-negative bacteria, while group 2 pumps, such as *tetK* and *tetL*, are significant in Gram-positive bacteria [60–62]. Tetracycline resistance proteins, encoded by genes like *tetM*, *tetL*, and *tetK*, protect bacterial ribosomes from tetracycline’s inhibitory actions. These genes are located on mobile genetic elements, facilitating horizontal gene transfer among bacterial species [63].

Erythromycin and clindamycin resistance were prevalent among the isolates. The absence of the *ermB* gene, coupled with a high prevalence of *ermA* in this study, suggests that macrolide resistance is primarily mediated by the *ermA* gene through ribosomal methylation [64]. A study from Egypt involving nasal swabs from 196 young volunteers found that 17.35% were colonized by *S. aureus*, while 35.20% carried other staphylococcal species. Notably, 50% of isolated *S. epidermidis* exhibited multidrug resistance, with a high prevalence of the *ermB* gene in both *S. aureus* (79.41%) and other staphylococcal species [65].

Macrolide efficacy is often compromised by Erm-type rRNA methyltransferase-mediated dimethylation at nucleotide A2058 on 23S rRNA, which is crucial for macrolide binding. High-resolution crystal structures showing interactions between Erm-dimethylated and undimethylated 70S ribosomes with macrolides have provided new insights that challenge previous resistance models. These findings reveal a novel role for the desosamine moiety in drug binding, potentially guiding future macrolide designs to overcome Erm-mediated resistance [64].

The presence of both *msrA/B* and *mecA* genes is associated with MRSA. The *mecA* gene encodes PBP2a, which has a low affinity for β-lactam antibiotics, conferring significant resistance to this drug class [66].

Biofilm formation, a major virulence factor of *S. epidermidis*, was observed in varying degrees among the isolates, with most demonstrating weak to moderate biofilm formation, while one isolate exhibited strong biofilm capability. This finding is clinically significant, as biofilms protect bacteria from host immune responses and antibiotic treatments, complicating infection management. The presence of biofilm-associated genes, such as *icaA* and *icaD*, correlates with the biofilm-forming abilities of the isolates. A positive correlation between biofilm formation and the presence of the *icaA* gene further supports its critical role in biofilm development.

Additionally, strong correlations between biofilm-associated genes, such as *arcA* and *opp3AB*, and *icaA* and *opp3AB*, suggest a complex regulatory network influencing biofilm formation and persistence in these isolates. Previous studies have shown that isookanin effectively inhibits biofilm formation, reducing biofilm mass by over 85% at a concentration of 250 mg/mL. Mechanistic analyses indicated that isookanin decreases the production of exopolysaccharides, eDNA, and surface hydrophobicity, while modulating essential biofilm-associated genes like *icaB*, *icaR*, and *RNAIII*. This enhances the efficacy of β-lactam antibiotics through synergistic interactions, as demonstrated by a favorable FICI index [67].

The presence of IS256 suggests a robust capacity for biofilm development, which may facilitate *S. epidermidis* colonization within the nasal cavity and its involvement in healthcare-associated infections [68]. IS256, a prevalent insertion sequence, is implicated in genomic rearrangements and the propagation of antibiotic resistance genes. The *arcA* and *opp3AB* genes contribute to bacterial survival by participating in arginine catabolism and peptide transport, respectively [69]. The *icaA* and *icaD* genes play key roles in *S. epidermidis* biofilm formation, encoding enzymes needed for the synthesis of PIA, which is critical for biofilm structure and bacterial persistence on surfaces [34]. The identification of the *icaA/D* genes along with IS256 sequence elements strongly correlates with treatment failures associated with coagulase-negative staphylococcal infections [70].

## Conclusion

This study highlights the significant antimicrobial resistance and biofilm-forming potential of nasal carriage *S. epidermidis* isolates. The high prevalence of MDR isolates, particularly those harboring both *erm* and *tet* genes, raises concerns about the potential for these commensal organisms to serve as reservoirs of resistance genes. Additionally, the biofilm-forming ability of certain isolates complicates treatment strategies and emphasizes the need for continuous surveillance of nasal carriage *S. epidermidis* in both healthy populations and clinical environments. These findings underscore the dual role of *S. epidermidis* as both a commensal organism and a potential pathogen in nosocomial infections. Future research should focus on elucidating the regulatory mechanisms that govern biofilm formation and exploring innovative strategies to combat biofilm-associated infections in clinical settings. Understanding these mechanisms is crucial for developing effective interventions to mitigate the impact of *S. epidermidis* in healthcare-associated infections.
